# Activation of the sigma-1 receptor exerts cardioprotection in a rodent model of chronic heart failure by stimulation of angiogenesis

**DOI:** 10.1186/s10020-022-00517-1

**Published:** 2022-08-03

**Authors:** Xin Zhao, Xin Liu, Xiuhuan Chen, Xueyu Han, Yazhou Sun, Yuhong Fo, Xiukun Wang, Chuan Qu, Bo Yang

**Affiliations:** 1 Department of Cardiology, Renmin Hospital of Wuhan University, 238 Jiefang Road, Wuchang District, Wuhan, 430060 Hubei People’s Republic of China; 2 Cardiovascular Research Institute, Wuhan University, 238 Jiefang Road, Wuchang District, Wuhan, 430060 People’s Republic of China; 3 Hubei Key Laboratory of Cardiology, Wuhan, 430060 People’s Republic of China

**Keywords:** The sigma-1 receptor, Heart failure, Angiogenesis, JAK2/STAT3

## Abstract

**Background:**

Angiogenesis plays a critical role on post-infarction heart failure (PIHF), the presence of which facilitates additional blood supply to maintain the survival of residual cardiomyocytes. The sigma-1 receptor (S1R) has been substantiated to stimulate angiogenesis, with the effect on a model of PIHF remaining unknown.

**Aims:**

This study aims to investigate the effects of S1R on PIHF and the underlying mechanisms involved.

**Methods:**

Rats were implemented left anterior descending artery ligation followed by rearing for 6 weeks to induce a phenotype of heart failure. Daily intraperitoneal injection of S1R agonist or antagonist for 5 weeks was applied from 2nd week after surgery. The effects exerted by S1R were detected by echocardiography, hemodynamic testing, western blot, Sirius red dyeing, ELISA, immunohistochemistry and fluorescence. We also cultured HUVECs to verify the mechanisms in vitro.

**Results:**

Stimulation of S1R significantly ameliorated the cardiac function resulted from PIHF, in addition to the observation of reduced fibrosis in the peri-infarct region and the apoptosis of residual cardiomyocytes, which were associated with augmentation of microvascular density in peri-infarct region through activation of the JAK2/STAT3 pathway. We also indicated that suppression of JAK2/STAT3 pathway by specific inhibitor in vitro reversed the pro-angiogenic effects of S1R on HUVECs, which further confirmed that angiogenesis, responsible for PIHF amelioration, by S1R stimulation was in a JAK2/STAT3 pathway-dependent manner.

**Conclusion:**

S1R stimulation improved PIHF-induced cardiac dysfunction and ventricular remodeling through promoting angiogenesis by activating the JAK2/STAT3 pathway.

**Supplementary Information:**

The online version contains supplementary material available at 10.1186/s10020-022-00517-1.

## Introduction

Heart failure (HF) is defined as a terminal stage of developmental cardiac disease, at which heart cannot pump adequate blood to various organs to meet the physiological demands ascribed to systolic and/or diastolic dysfunction (Meer et al. 2019). Heart failure involves the activation of multiple endogenous neuroendocrine and cytokines, and the long-term activation contributes to myocardial damage and ventricular remodeling, while current therapies do not completely reverse these pathophysiological alterations (Liang et al. 2020). With the increase of global aging population, the incidence of heart failure has increased year by year (Roger 2013). HF is a primary health burden globally. For example, in China, 4.5 million people are currently suffering from HF, with a prevalence of approximately 1.9% (Weiwei et al. 2016). Despite the significant development of HF pathophysiological research and therapeutic modalities, HF is still one of the major diseases threatening the safety and health of human all over the world (Savarese and Lund 2017).

Myocardial infarction (MI) remains a dominant cause of HF, which often leads to myocardial apoptosis, interstitial fibrosis and cardiomyocyte hypertrophy, all of these might contribute to ventricular remodeling and cardiac dysfunction (Jenča et al. 2021; Gabriel-Costa 2018). After MI, myocardial tissue is severely ischemic and hypoxic, where the generation of reactive oxygen species (ROS), infiltration of pro-inflammatory cytokines, and the loss of normal nutritional support might elicit endothelial cell injury in and around the infarcted area (Wu et al. 2021), giving rise to dramatical microvascular density rarefaction, which further results in peripheral myocardial necrosis and fibrosis, followed by decline in cardiac function and initiation of HF (Spears 2019). Endothelial cells are generally dormant, with the ability to initiate vasoformation retained, a process known as angiogenesis to supply blood flow with oxygen and nutrients, which could be activated in an ischemic and hypoxic milieu (Eelen et al. 2020; Tabibiazar and Rockson 2001), therefore, angiogenesis played a crucial role on MI and HF. A consensus remains that promotion of angiogenesis to increase capillary and arteriovenous density in a murine model of MI, could reduce cardiac fibrosis and improve post-infarction cardiac function, eventually delaying the pathological transition to HF (Moghiman et al. 2021). Therefore, angiogenesis is a promising approach to ameliorate post-infarction HF. However, there is a vast gap between basic research and clinical trials to confirm the safety and efficacy of pro-angiogenic drugs to date (Beohar et al. 2010).

The sigma-1 receptor (S1R) was initially proposed as a nonopioid receptor expressed in many organs such as the brain, retina, liver, and lung, with another abundance in the heart. Subsequent studies found that S1R is an endoplasmic reticulum (ER) transmembrane chaperone protein, mainly located in the mitochondria-associated ER membrane (MAM), which can regulate multiple cellular functions (Hayashi and Su 2007). The S1R serves cardioprotective functions as an intercellular organelle signaling molecule. Moreover, accumulating studies have demonstrated that stimulation of S1R could prevent cardiac functional deterioration induced by progress in cardiac remodeling. Deletion of S1R in S1R^−/−^ mouse models leads to mitochondrial dysfunction and cardiac remodeling, followed by gradual progress to cardiac dysfunction (Abdullah et al. 2018). Several selective serotonin reuptake inhibitors (SSRIs) and tricyclic antidepressants have distinct affinity for S1R, among which fluvoxamine (FLV) that is associated with reduction of the incidence of post-infarction depression and improvement of the prognosis of coronary artery disease (Hashimoto 2013) has the most substantial effect. Previous studies have shown that FLV inhibits Ca^2+^ transporter-Type 2 inositol 1,4,5-trisphosphate 6 receptors (IP3R2) expression through stimulation of S1R, which leads to repair of impaired calcium cycling and elevates energy metabolism in rats with MI (Lou et al. 2021). Our earlier study also indicated that stimulation of S1R by FLV improved cardiac function by reducing sympathetic neurogenesis and myocardial fibrosis (Fo et al. 2020). However, the role for S1R on cardiac angiogenesis responsible for the alleviation of ventricular remodeling remains unknown. We hypothesized that stimulation of S1R may prevent HF by improving angiogenesis in reasonable consideration of the pleiotropic effects exerted by S1R on cardiac remodeling.

Therefore, this article aimed to investigate whether chronic stimulation of S1R could improve left ventricular remodeling and cardiac function in rats with post-infarction HF and explore the possible mechanisms.

## Methods and materials

### Animal models and treatments

All experiments were authorized by the Ethics Committee of the Renmin Hospital of Wuhan University (WDRM20210906) and were conducted under the guidance of the Care and Use of Laboratory Animals published by the US National Institutes of Health (NIH Publication, 2011). Male Sprague‑Dawley (SD) rats (age 6–7 weeks; weight 200–220 g) were purchased from Beijing Vital River Laboratory Animal Technology Co., Ltd.

1 week after adaptive housing of rats, all animals underwent MI surgery or sham surgery. All rats were anesthetized with 3% pentobarbital sodium (2 ml/kg, Sigma-Aldridge, intraperitoneal injection) and intubated with a tracheal tube to support breath, except for the sham-operated group, in which only the heart was threaded without ligation, the remaining rats underwent thoracotomy followed by the left coronary artery (LAD) ligation and closed the chest, and the successful MI model was confirmed by the ST-segment elevation on the ECG recorder or occurrence of the paleness of the anterior ventricular region distal to the ligature. All rats were given intramuscular penicillin (200,000 IU/day) postoperatively for 1 week.

PIHF model made by continuous rearing for 6 weeks after surgery. The rats were randomized into four groups: (a) sham + saline (sham group, n = 10); (b) MI + saline (HF group, n = 10); (c) MI + FLV (HF + F group, n = 10); (d) MI + FLV + BD1047 group (HF + F + BD group, n = 10). FLV (0.3 mg/kg/day, Sigma-Aldridge) and BD1047 (0.3 mg/kg/day, Sigma-Aldridge) were injected intraperitoneally, and the remaining two groups received equivalent volume of 0.9% saline. The sigma-1 receptors can be selectively antagonized by BD-1047 (HY-16996A), while fluvoxamine (FLV, HY-B0103) has an agonistic effect on it. The chemicals were purchased from MedChemExpress (MCE).

### Echocardiography measurements

After anesthetizing rats with isoflurane gas, cardiac function indicators were measured by echocardiography at the 6th week after surgery. Left ventricular end-diastolic internal diameter (LVIDd), left ventricular end-systolic inner diameters (LVIDs), left ventricular systolic percentage (FS), left ventricular ejection fraction (LVEF), left ventricular posterior wall diastole (LVPWd) were recorded for at least three consecutive cycles for data analysis.

### Measurement of hemodynamic parameters

Hemodynamic parameters were measured six weeks after left coronary artery ligation. The rats were anesthetized and fixed on the operating table, and the right common carotid artery was separated through the mid-cervical incision. A catheter (PE50) filled with heparin saline, which was connected to the pressure converter, was inserted into the carotid artery and advanced into the left ventricle (LV). The blood pressure would be displayed in the computer through the pressure converter, when the pressure amplitude increased and the lowest point could reach 0, proving the catheter had entered the left ventricle (Fraccarollo et al. 2003). Then measured the LV systolic pressure (LVSP), LV end-diastolic pressure (LVEDP), maximal rate of pressure rise and decline (dP/dt_max_ and dP/dt_min_) using the PowerLab system (AD Instruments), recording these use LabChart 8.0 software.

### ELISA

After anesthetizing the rats (n = 6, per group), 5 ml blood was collected via the inferior vena cava using an EDTA-K_2_ in evacuated tubes and centrifuged at 3000 g for 15 min, 4 °C (Beckman Coulter, USA). The plasma was lyophilized by dry ice and temporarily stored in a − 80 °C refrigerator to prevent degradation of the active substance. N-terminal pro-B type brain natriuretic peptide (NT-proBNP) concentration in serum was estimated by ELISA. The ELISA kit (FB-N06148R) was purchased from Wuhan Fengbin Technology Co., Ltd.

### SR and IHC staining

The 4% paraformaldehyde-fixed heart tissue was embedded in paraffin to make 4-μm sections. Heart sections were stained with Sirius Red (SR). Neovascularization refers to the sprouting of new microvessels from pre-existing coronary arteries, defined as vessels less than 400 μm in diameter (Chang et al. 2021; Cochain et al. 2013). Angiogenesis in cardiac tissue was observed by immunohistochemistry (IHC). The sections were dewaxed with xylene and then rehydrated using different ethanol gradients. After antigen repair by thermal repair, the sections were incubated with 3% hydrogen peroxide solution for endogenous peroxidase blocking, CD31 antibody (Abcam, ab182981, 1:1000) was added and incubated overnight at 4 degrees C. After washing, the sections were incubated with a secondary antibody (DAKO, K5007) for 50 min at room temperature, The sections were developed using DAB color developer (DAKO, K5007, 1:100), and rinsed to stop color development when the positive area was brownish yellow. After antigen repair, the antibody incubation step is repeated using PCNA (Abcam, ab92552, 1:250) as the primary antibody. Eventually, the nuclei were re-stained with hematoxylin, dehydrated and sealed. α-smooth muscle actin (α-SMA) (Boster, BM0002, 1:200) was also stained immunohistochemically, which is used to label pericytes and vascular smooth muscle cells around the vascular endothelium. Lastly, images were recorded using digital scanning (Pannoramic 250/MIDI), and data analysis was performed using Image J software (NIH, Bethesda, MD, USA) to calculate collagen deposition, infarct areas and neovascularization density.

The heart tissue sections were washed three times in PBS and closed with serum for 30 min at room temperature. S1R antibody (Abcam, ab253192, 1:500) was added and incubated overnight at 4 degrees C. After washing, the sections were incubated with a secondary antibody (Jackson, 115-165-003, 1:200) for 50 min at room temperature in the dark. Finally, the nuclei were re-stained using DAPI (Solarbio, 0100-100, 1:100) and incubated for 10 min avoiding light. The sections were placed under a fluorescent microscope for observation and image acquisition.

### Terminal deoxynucleotidyl transferase nick end–labeling (TUNEL) assay

Using the heart sections for TUNEL testing, follow the steps of the TUNEL immunofluorescence kit (Roche, Switzerland; 11684817910) to perform the experiment. In the final stage, the images were observed under a fluorescent microscope, and the apoptosis rate of cardiomyocytes was calculated using Image J software.

### HUVECs culture and treatment in vitro

Using the DMEM/HIGH GLUCOSE medium (HyClone) containing 10% fetal bovine serum (FBS, LONSERA) to culture human umbilical vein endothelial cells (HUVECs). Cells were incubated in humid air containing 5% CO_2_ at 37 °C. HUVECs were inoculated in 6-well plates and when the cell density reached 80%, transfected with Ad-S1R and Ad-control, using a viral titer of MOI = 20, incubated for 6 h. Subsequently, the culture medium containing the virus was replaced using complete medium. After 24 h, the cells were cultured for 48 h using a medium containing 50 μM isoproterenol (ISO) to imitate the HF model (Wang et al. 2021). Dividing the cell experiment into two parts, part one: (a) control group; (b) ISO medium group; (c) ISO medium + Ad-control group; (d) ISO medium + Ad-S1R group; (e) ISO medium + Ad-S1R + 5 μM BD1047 group (Vahabzadeh et al. 2020). Part two: (a) control group; (b) control medium + Ad-S1R group; (c) control medium + Ad-S1R + 50 μM AG490 group (Zhao et al. 2018); (d) ISO medium group; (e) ISO medium + Ad-S1R group (e) ISO medium + Ad-S1R + 50 μM AG490 group. Part three: (a) ISO medium group; (b) ISO medium + FLV (5 μM) group (Tagashira et al. 2014) (c) ISO medium + Ad-S1R group. HUVECs were purchased from Wuhan Huaer Biotechnology Co., LTD. The chemical inhibitors were purchased from MedChemExpress (MCE). Both adenoviruses were constructed by GV314 vector, BamHI/AgeI digestion, purchased from Shanghai GeneCyte Chemical Technology Co.

### Tube-formation and cell viability assay

50 μL of matrix gel (BD Biosciences) was added to the 96-well plate, and after it solidified, 100 μL treated cell suspension (1 × 10^4^ cells/mL) was seeded into the 96-well plate. After six hours, the angiogenesis of HUVECs was observed under an inverted light microscope and photographed, the tube lengths were calculated using Image J software. We also used a cell counting kit-8 (CCK-8) to detect cell viability. When the cell density reached 1 × 105 cells per well, 10 μL of the cck-8 solution was added and incubated for 2 h. The absorbance at 450 nm was measured with an enzyme marker.

### Western blot

The proteins were extracted from the tissue of the peri-infarct area of rats and treated HUVECs, then quantified by the BCA protein assay kit. The membranes were incubated with antibodies against S1R (1:1000, ab253192, Abcam), JAK2 (1:1000, ab108596, Abcam), phosphorylated JAK2 (1:1000, ab32101, Abcam), STAT3 (1:1000, ab68153, Abcam), phosphorylated STAT3 (1:1000, ab267373, Abcam), VEGF (1:1000, ab214424, Abcam), bcl2 (1:1000, ab182858, Abcam), bax (1:1000, ab32503, Abcam), cleaved caspase-3 (1:1000, ab2302, Abcam), TGF-β1 (1:1000, ab215715, Abcam), phosphorylated VEGFR2 (1:1000, ab5473, Abcam) and GAPDH (1:1000, GB15004, Servicebio).Then, these were incubated with HRP-conjugated secondary antibodies (1:3000, GB23303, GB23301, Servicebio). The western blot images were then analyzed and calculated using Image J software.

### Statistical analysis

Continuous variables were shown as mean ± SD and proportions were shown as percentages. The Kaplan–Meier method was applied to analyze survival. Differences between groups were compared using one-way analysis of variance (ANOVA) and corrected by Tukey post hoc test. Statistically significant was considered P < 0.05.

## Results

### Chronic stimulation of S1R modified cardiac function in PIHF rats

**Fig. 1:**
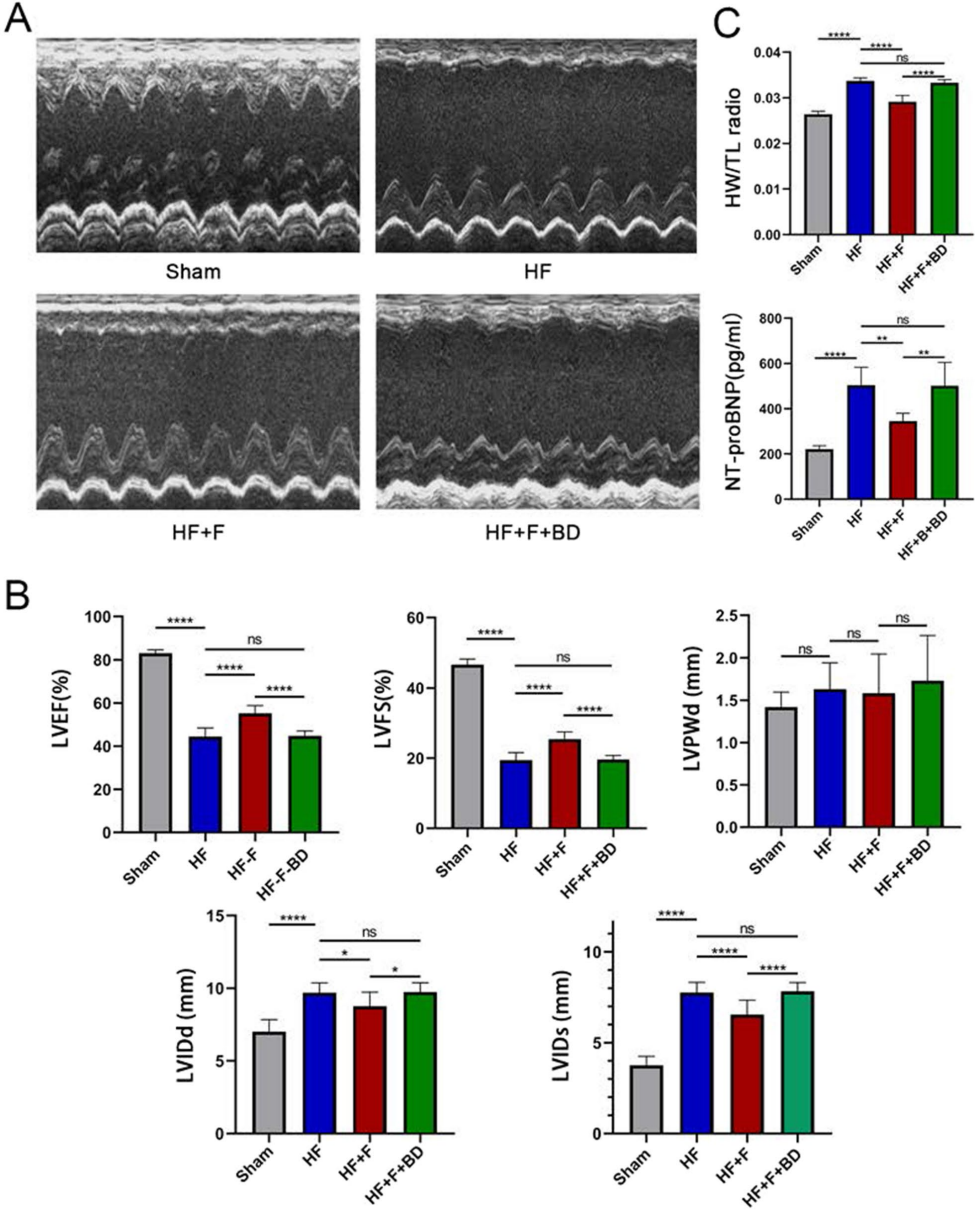
The effects exerted by chronic stimulation of the sigma-1 receptor on cardiac functions. **A** Representative M-mode echocardiogram images in all groups; **B** Serum BNP concentration and heart weight/tibial length, reflecting the severity of heart failure and cardiac hypertrophy; **C** Statistical analysis of parameters relative to cardiac function; *P < 0.05; **P < 0.01; ***P < 0.005; ****P < 0.001

**Fig. 2:**
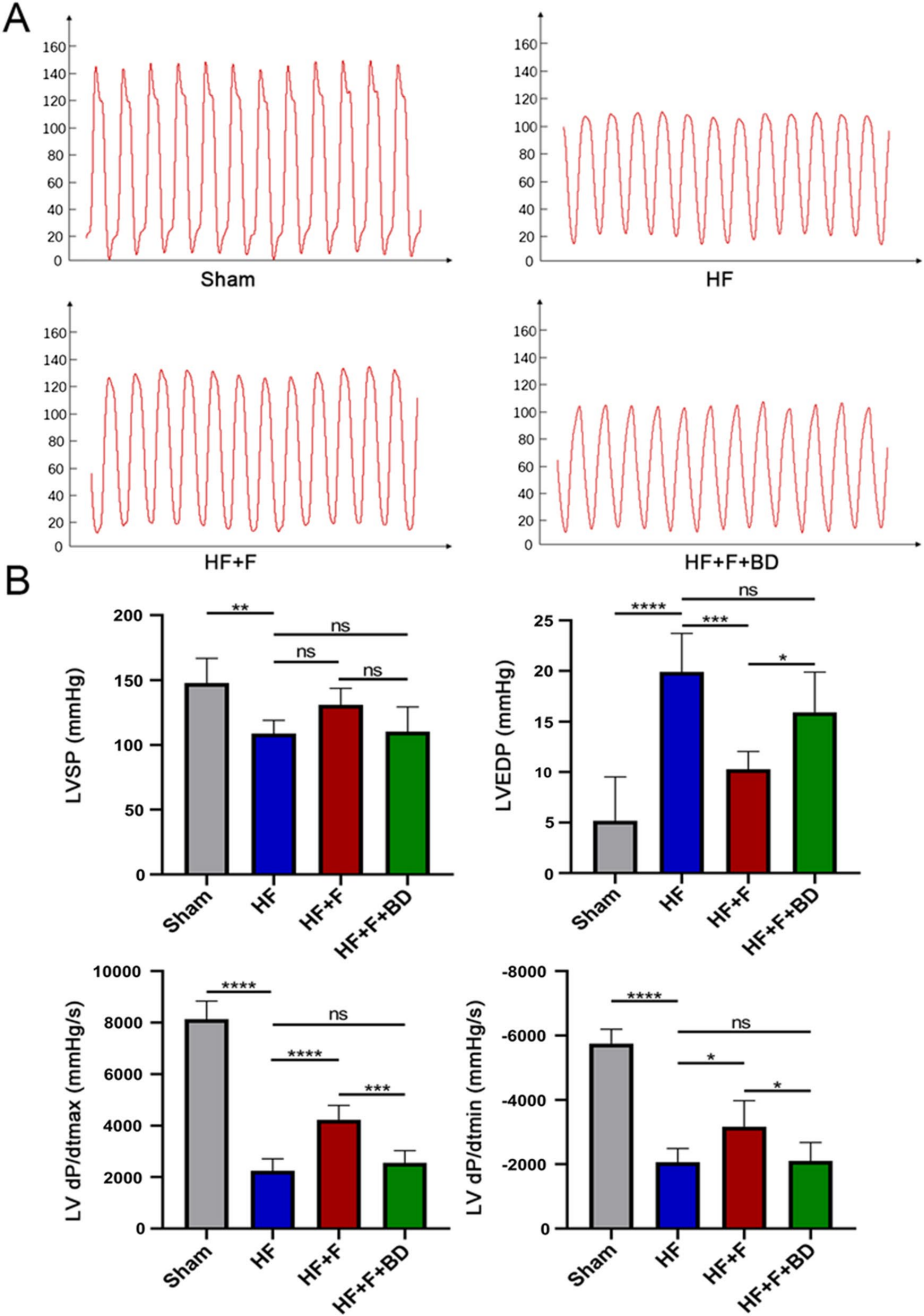
Stimulation of sigma-1 receptor enhanced hemodynamics in PIHF rats. **A** Representative left ventricular pressures images in all groups; **B** LVSP, LVEDP, LV dP/dt_max_, and LV dP/dt_min_ in rats 6 weeks after MI or sham operation. *P < 0.05; **P < 0.01; ***P < 0.005; ****P < 0.001

Echocardiography was implemented to evaluate the cardiac function at 6th week after surgery. In contrast to the sham group, rats conducted LAD ligation showed defective cardiac dysfunction, embodied by dramatical decrease of LVEF (44.48 ± 3.95 vs. 83.10 ± 1.57%, *P* < 0.001) and FS (19.41 ± 2.17 vs. 46.67 ± 1.58%, *P* < 0.001), accompanied by increase of LVIDd (9.70 ± 0.67 vs. 7.02 ± 0.82 mm, *P* < 0.001) and LVIDs (7.76 ± 0.56 vs. 3.75 ± 0.50 mm, *P* < 0.001), but not LVPWd (1.63 ± 0.31 vs. 1.42 ± 0.18 mm, P = 0.64, Fig. 1A and B). Furthermore, hemodynamics and LV function indicators, detected by left ventricular catheter insertion, also revealed detrimental oscillations in ventricular systole and diastole in PIHF group rats versus sham rats, where the LVSP (108.90 ± 10.17 vs. 147.80 ± 18.98 mmHg, *P* < 0.01), dP/dt_max_ (2242 ± 472.50 vs. 8138 ± 702.50 mmHg/s, *P* < 0.001), and dP/dt_min_ (− 2063 ± 429.20 vs. − 5753 ± 446.70 mmHg/s, *P* < 0.001) were significantly decreased, while LVEDP (19.91 ± 3.80 vs. 5.18 ± 4.35 mmHg, *P* < 0.001), in turn, was increased (Fig. 2A and B). In addition, NT-proBNP concentration in serum (504.20 ± 78.88 vs. 220.80 ± 15.37 pg/ml, *P* < 0.001) and heart weight/tibial length (0.034 ± 0.0007 vs. 0.026 ± 0.0007, *P* < 0.001) were observably augmented in this group of rats (Fig. 1C). These results synergistically demonstrated the successful establishment of PIHF. However, intraperitoneal administration of FLV, which is known as an extensively used S1R agonist, improved cardiac function, encompassing echocardiographic and hemodynamic indices, serum NT-proBNP concentration (344.70 ± 35.04 vs. 504.20 ± 78.88 pg/ml, *P* < 0.01), and cardiac hypertrophy, namely, heart weight/tibial length (0.029 ± 0.0013 vs. 0.034 ± 0.0007, *P* < 0.001), in HF + F group rats, indicating that stimulation of S1R was sufficient to ameliorate the manifestations of PIHF. All these effects exerted by S1R stimulation were counteracted in those rats simultaneously injected with FLV and S1R antagonist, BD1047, further verifying the role for S1R on PIHF. The summary of the parameters was illustrated in Tables 1 and 2.

**Table 1:** Cardiac function parameters of the left ventricle

	LVEF	LVFS	LVIDd	LVIDs	LVPWd
	(%)	(%)	(mm)	(mm)	(mm)
Sham	83.10 ± 1.57	46.67 ± 1.58	7.02 ± 0.82	3.75 ± 0.50	1.42 ± 0.18
HF	44.48 ± 3.95^††††^	19.41 ± 2.17^††††^	9.70 ± 0.67^††††^	7.76 ± 0.56^††††^	1.63 ± 0.31
HF + F	55.25 ± 3.60****	25.46 ± 2.08****	8.77 ± 0.96*	6.56 ± 0.79****	1.58 ± 0.46
HF + F + BD	44.80 ± 2.24^####^	19.59 ± 1.18^####^	9.74 ± 0.65^#^	7.83 ± 0.49^####^	1.73 ± 0.53

LVEF, left ventricular ejection fraction; LVFS, left ventricular systolic percentage; LVIDd, left ventricular end-diastolic internal diameter; LVIDs, left ventricular end-systolic inner diameters; LVPWd, left ventricular posterior wall diastole; ^†^P HF group vs sham group; *P HF + F group vs. HF group; ^#^P HF + F + BD group vs HF + F group. *^,^^#^: *P*<0.05; ^††††, ^****^, ####^: *P*<0.001

**Table 2:** Parameters of hemodynamics, cardiac structure, and NT-proBNP

	HW/TL	NT-proBNP	LVSP	LVEDP	LV dP/dt_max_	LV dP/dt_min_
	Ratio	(pg/ml)	(mmHg)	(mmHg)	(mmHg)	(mmHg)
Sham	0.026 ± 0.0007	220.8 ± 15.37	147.8 ± 18.98	5.18 ± 4.35	8138 ± 702.5	− 5753 ± 446.7
HF	0.034 ± 0.0007^††††^	504.2 ± 78.88^††††^	108.9 ± 10.17^††^	19.91 ± 3.80^††††^	2242 ± 472.5^††††^	− 2063 ± 429.2^††††^
HF + F	0.029 ± 0.0013****	344.7 ± 35.04**	131.1 ± 12.61	10.29 ± 1.77***	4226 ± 564.1****	− 3170 ± 807.1*
HF + F + BD	0.033 ± 0.0007^####^	501.9 ± 103.10^##^	110.4 ± 19.05	15.92 ± 3.96^#^	2557 ± 472.8^###^	− 2102 ± 573.4^#^

HW/TL, heart weight/tibial length; NT-proBNP, NT-proBNP concentration in serum; LVSP, LV systolic pressure; LVEDP, LV end-diastolic pressure; LV dp/dt_max_, LV dp/dt_min_, maximal rate of pressure rise and decline (dP/dt_max_ and dP/dt_min_); ^†^P HF group vs sham group; *P HF + F group vs. HF group; ^#^P HF + F + BD group vs HF + F group. *^, #^: P<0.05; ^††, ^**^, ##^: *P*<0.01; ***^, ###^: P<0.005; ^††††,^ ****^, ####^: *P*<0.001

### Chronic stimulation of S1R improved ventricular remodeling in PIHF rats

**Fig. 3:**
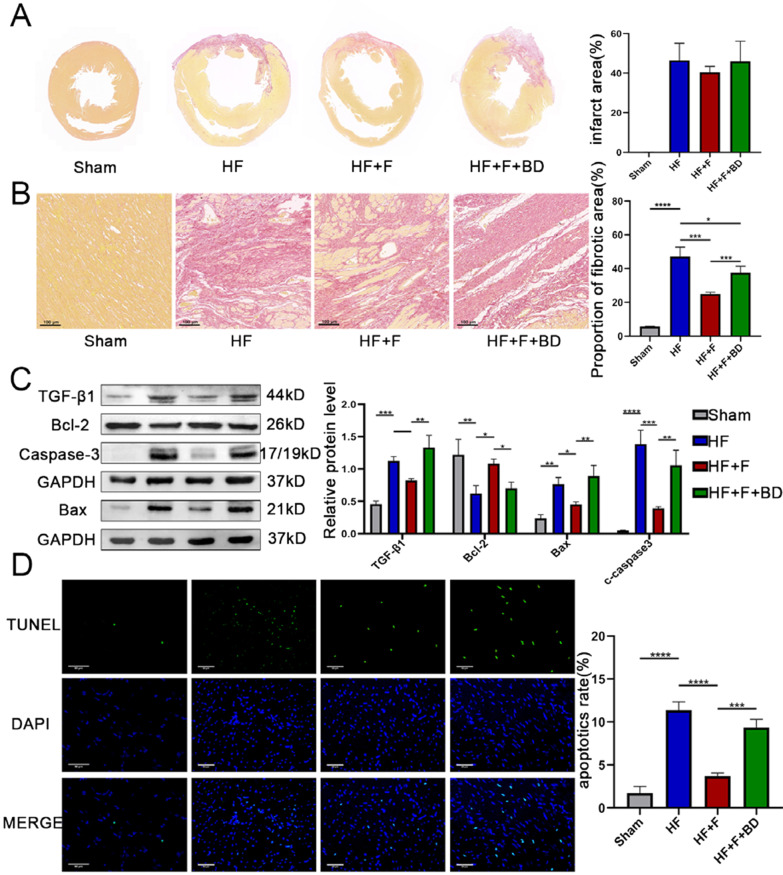
Stimulation of sigma-1 receptor alleviated post-infarct heart failure-induced ventricular remodeling. **A** S1R stimulation reduced infarct area; **B** S1R stimulation decreased collagen deposition in the peri-infarction zone, evidenced by the Sirius red dyeing; **C** Western blot results showed that stimulation of S1R improved apoptosis and fibrosis. N = 3 for quantified analysis; **D** S1R stimulation reduced cardiomyocyte apoptosis in the peri-infarct region, which was detected by Terminal-deoxynucleotidyl Transferase-Mediated Nick-End Labeling (TUNEL) assay. *P < 0.05; **P < 0.01; ***P < 0.005; ****P < 0.001

Albeit administration of FLV ameliorated cardiac function, a nominally reduction of infarct size was observed compared to the HF group (40.48 ± 3.01 vs. 46.36 ± 8.80%, *P* = 0.73), which might be attributed to a scarcity of regeneration of cardiomyocytes (Fig. 3A). We then exploited two remodeling indicators, namely, fibrosis and apoptosis to determine the alterations of ventricular structure. Excessive interstitial collagen deposited in the infarct-border zones of PIHF rats, which was significantly ameliorated by application of S1R agonist FLV (24.89 ± 0.95 vs. 47.19 ± 5.50%, *P* < 0.005, Fig. 3B), indicating anti-fibrosis effect of S1R, analogous to previous studies (Qu et al. 2021). Subsequently, we confirmed this observation by western blot experiment. TGF-β1 is a transforming growth factor with a strong pro-fibrotic activity to facilitate collagen secretion and crosslinking. Therefore, we conducted TGF-β1 detection, the alteration of which was consistent with pathological change of interstitial fibrosis (Fig. 3C and Fig. S2). In addition, stimulation of S1R mitigated PIHF-induced cardiomyocyte apoptosis in the ventricular. With regarding to that the pro-apoptotic gene Bax activates Caspase-3, leading to apoptosis, while the anti-apoptotic gene Bcl-2 inhibits apoptosis by blocking this process (Scarabelli et al. 2006), we performed western blot analysis, where the upregulation of anti-apoptotic gene bcl-2, in addition to the downregulation of pro-apoptotic bax and cleaved caspase-3 in the HF + F group validated anti-apoptotic role for S1R (Fig. 3C and Fig. S2). Further TUNEL analysis substantiated this effect, which was offset by S1R antagonist, in accordance with previous observations (3.68 ± 0.39 vs. 11.38 ± 0.97%, *P* < 0.001, Fig. 3D). In a conclusion, our results demonstrated that stimulation of S1R was associated with alleviation of ventricular remodeling in a model of PIHF.

### Angiogenesis stimulated by S1R as a mechanism for amelioration of cardiac function and remodeling

**Fig. 4:**
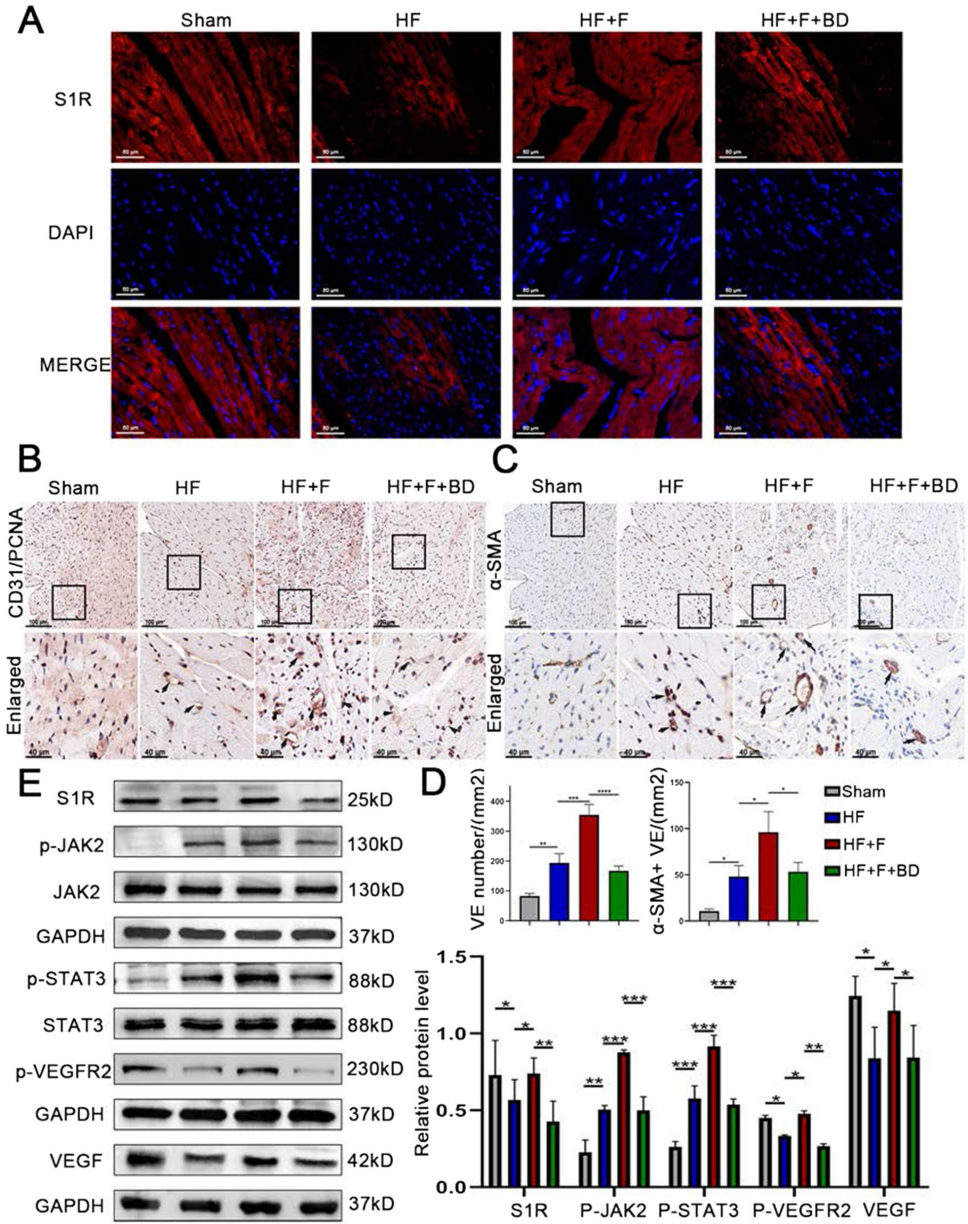
Stimulation of the sigma-1 receptor moderated the proangiogenic switch via JAK2/STAT3 signaling pathway in PIHF rats. **A** Immunofluorescence staining showing intracellular localization of S1R (red); **B** S1R stimulation ameliorated angiogenesis, evidenced by the CD31 and PCNA double immunohistochemical staining; **C** S1R stimulation promote mature vascular lumen generation by the α-sma immunohistochemical staining; **D** Quantitative analysis results of CD31 + and SMA + vessels; **E** Western blot results showed that stimulation of S1R upregulated the JAK2/STAT3 signaling pathway to pro-angiogenesis. N = 3 for quantified analysis. *P < 0.05; **P < 0.01; ***P < 0.005; ****P < 0.001

We further aimed to identify the mechanisms by which stimulation of S1R ameliorated cardiac function and remodeling. We firstly examined the distribution of S1R in cardiac tissues by immunofluorescence and the expression level of S1R by western blot. The S1R immunofluorescence intensity and protein expression were lower in the PIHF group than the sham group, opposite to a reversal by injection of S1R agonist FLV which significantly upregulated S1R (Fig. 4A, the top panel of Fig. 4E and Fig. S3), implying an association between pharmaceutical stimulation and upregulation of S1R. In consideration of the fact that accumulating studies have demonstrated that angiogenesis is the process of issuing new capillary branches from the original capillaries, followed by pericytes and vascular smooth muscle encapsulation to form a stable mature vascular lumen (Khurana et al. 2005), which could exert prominent protective effect on cardiac remodeling, therefore, we executed CD31/PCNA double and α-SMA immunostaining, the analysis of which indicated the vascular density in the peri-infarct region, to investigate the effect of stimulation of S1R on angiogenesis. CD31 immunostaining results indicated that stimulation of S1R increased microvascular density in the peri-infarct region (354.70 ± 34.49 vs. 193.30 ± 31.07 number/mm^2^, *P* < 0.01), with which co-localization of PCNA staining ascribed this increase to concomitant proliferation of endothelial cells, and the presence of mature vessels, as shown by α-SMA immunostaining (88.67 ± 8.62 vs. 48.33 ± 7.77 number/mm^2^, *P* < 0.005), was also indicative of an augmented blood supply to residual cardiomyocytes, thus counteracting PIHF-induced local ischemia (Fig. 4B and C). Afterwards, we aimed to explore the underlying mechanisms that S1R facilitated angiogenesis. Previous studies have confirmed that the STAT3 signaling pathway was responsible for angiogenesis to protect the myocardium (Chen and Han 2008). Western blot results showed that stimulation of S1R in HF + F rats promoted phosphorylation of JAK2 and STAT3 markedly (Robson et al. 2014), without any alterations on total JAK2 and STAT3, which further upregulated downstream p-VEGFR2 and VEGF expression (Fig. 4Eand Fig. S3). However, all of the above changes induced by S1R diminished with the application of S1R antagonist. Taken together, these results demonstrated that stimulation of S1R might ameliorate PIHF through activation of JAK2/STAT3 signaling pathway responsible for supplementary angiogenesis.

### S1R promote proliferation and tube formation of HUVECs in vitro under HF conditions

**Fig. 5:**
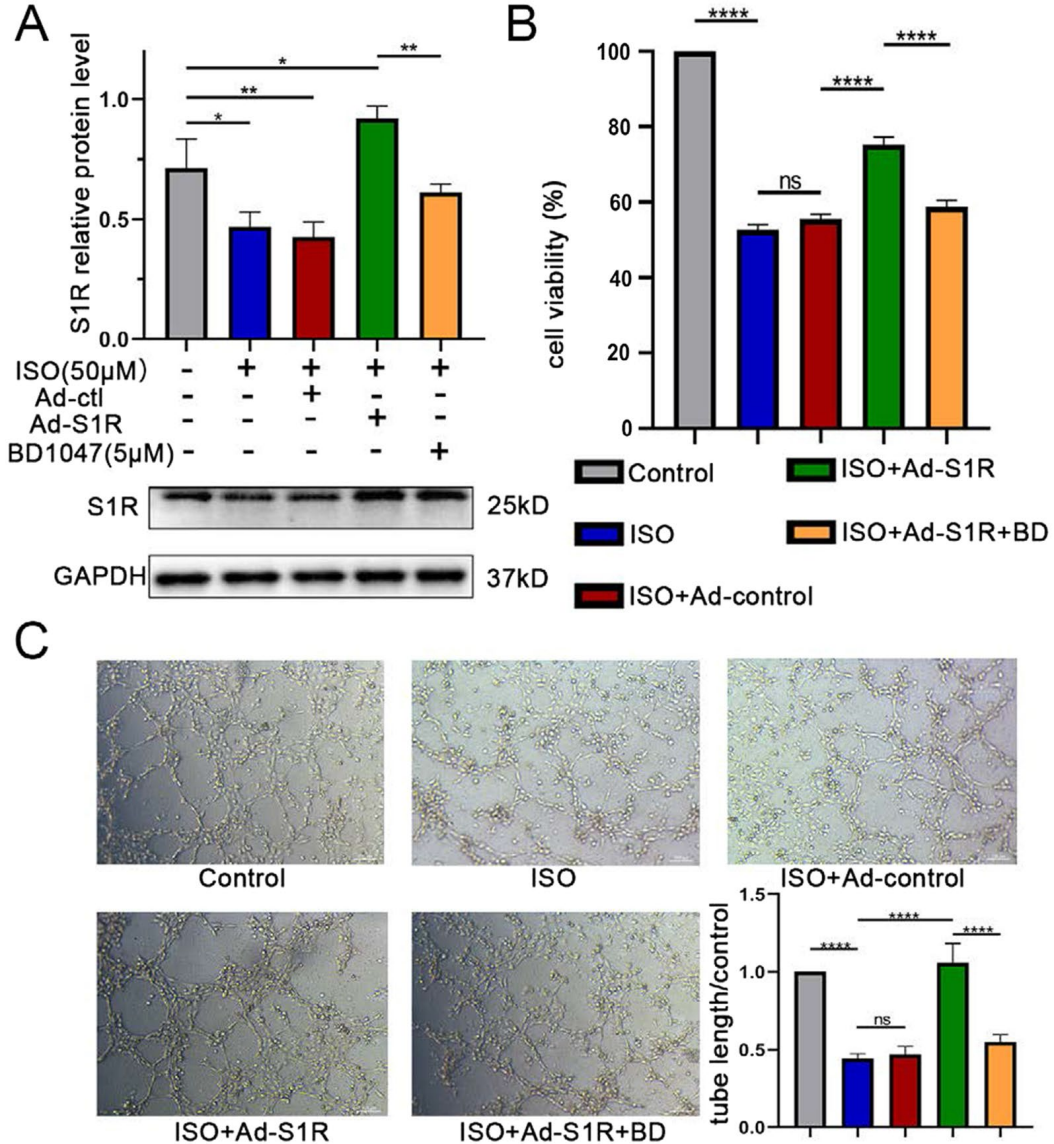
Transfection of Ad-S1R promoted angiogenesis of HUVECs. **A** Western blot results showed that transfection of AAV-S1R upregulated the sigma-1 receptor expression. N = 3 for quantified analysis; **B** Ad-S1R transfected enhanced proliferation of HUVECs, confirmed by CCK-8 experiment; **C** Ad-S1R transfected HUVECs accelerated tube formation. *P < 0.05; **P < 0.01; ***P < 0.005; ****P < 0.001

We next implemented in vitro experiments to verify the effects of S1R on angiogenesis. HUVECs were cultured using the medium containing 10% serum with 50 μM ISO to mimic the pathological status of HF in vitro. Based on previous results, stimulation of S1R also upregulated the expression of S1R, we thus utilized Ad-S1R to transfect HUVECs to induce S1R overexpression. The S1R expression was inhibited in the ISO-treated group, which was, in turn, upregulated by transfection with Ad-S1R (Fig. 5A and Fig. S4). We subsequently used cell proliferation and tube-formation assays to assess angiogenic ability (Duan et al. 2015). Angiogenic ability was inhibited in the ISO group versus the control group (0.45 ± 0.03 vs. 1.00 ± 0.02, *P* < 0.001), but these characteristics were recovered after transfection with Ad-S1R (1.05 ± 0.17 vs. 1.00 ± 0.02, *P* < 0.001). There were no differences in angiogenic capacity after coincubation of ISO and Ad-control versus control disposal (0.47 ± 0.06 vs. 0.45 ± 0.03, *P* = 0.99), suggesting insufficient impact of adenovirus vector on the results of this experiment. Analogously, the pro-angiogenic effect of Ad-S1R was reversed by BD-1047 (0.51 ± 0.03 vs. 1.05 ± 0.17, *P* < 0.001, Fig. 5B and C, Additional file 1: Fig. S7 and Table 2). Moreover, in consideration of the fact that absolute overexpression of S1R by transfection with Ad-S1R might distinguish with pharmaceutical stimulation to relative upregulation, we reestablished another cellular model to detect effects of FLV on tube-formation. Consistent with our anticipation, application of FLV reversed the detrimental effects of ISO (1.54 ± 0.08 vs. 1.00 ± 0.04, *P* < 0.005, Additional file 1: Fig. S1 and Fig. S6), suggesting that either overexpression or pharmaceutical stimulation of S1R could exert analogous promotion on angiogenesis.

### S1R activates the JAK2/STAT3 pathway in HUVECs

**Fig. 6:**
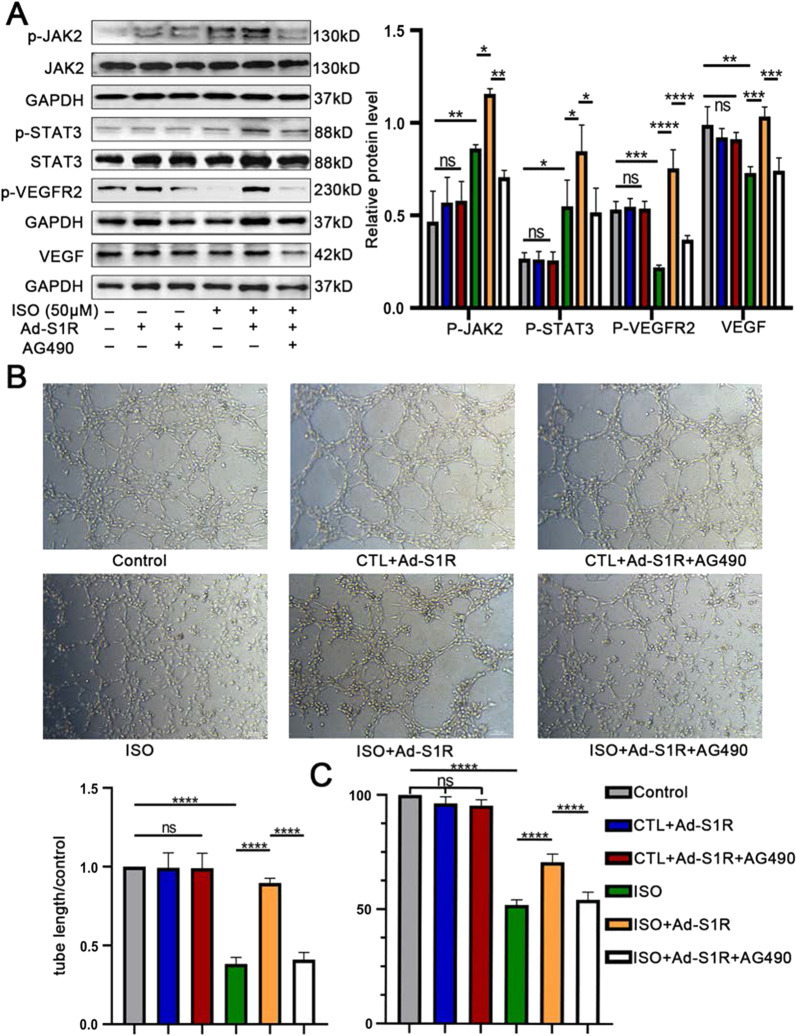
Transfection of Ad-S1R promoted angiogenesis of HUVECs through JAK2/STAT3 signaling pathway. **A** Western blot results showed that AG490 inhibited JAK2/STAT3 signaling pathway activation. N = 3 for quantified analysis; **B** In vitro incubation of JAK2/STAT3 inhibitor 50 μM AG490 decreased tube formation; **C** AG490 counteracted the pro-proliferative effect of Ad-S1R transfection on HUVECs; *P < 0.05; **P < 0.01; ***P < 0.005; ****P < 0.001

To further confirm the hypothesis that the beneficial effects of S1R on angiogenesis was JAK2/STAT3 pathway-dependent, rather than an improvement on integral pathological status or through other pro-angiogenic signaling pathway, we exploited AG490, the specific antagonist of the JAK2/STAT3 pathway (Bhuiyan et al. 2013). Interestingly, the JAK2/STAT3 pathway could be slightly activated as a compensatory mechanism upon HF status, and transfection with Ad-S1R could further increase phosphorylation of JAK2 and STAT3, followed by an upregulation of p-VEGFR2 and VEGF expression, all the effects of which, however, were counteracted by AG490. JAK2/STAT3 pathway, p-VEGFR2 and VEGF were not affected by Ad-S1R and AG490 under normal conditions (Fig. 6A and Fig. S5). In accordance with previous results, when HUVECs were transfected with Ad-S1R on HF-mimic condition, the cell proliferation and tube-forming ability could be facilitated versus those cells that were transfected with Ad-control. Moreover, in line with our speculation, incubation of AG490 nearly eliminated the efficacy of Ad-S1R (0.41 ± 0.03 vs. 0.90 ± 0.06, *P* < 0.001), unraveling the fact that S1R attributed, at least partly, improvements in angiogenesis to an activation of JAK2/STAT3 pathway. Likewise, there was no change on angiogenic ability under normal conditions (Fig. 6B and C, Additional file 1: Fig. S8 and Table 1).

## Discussion

**Fig. 7:**
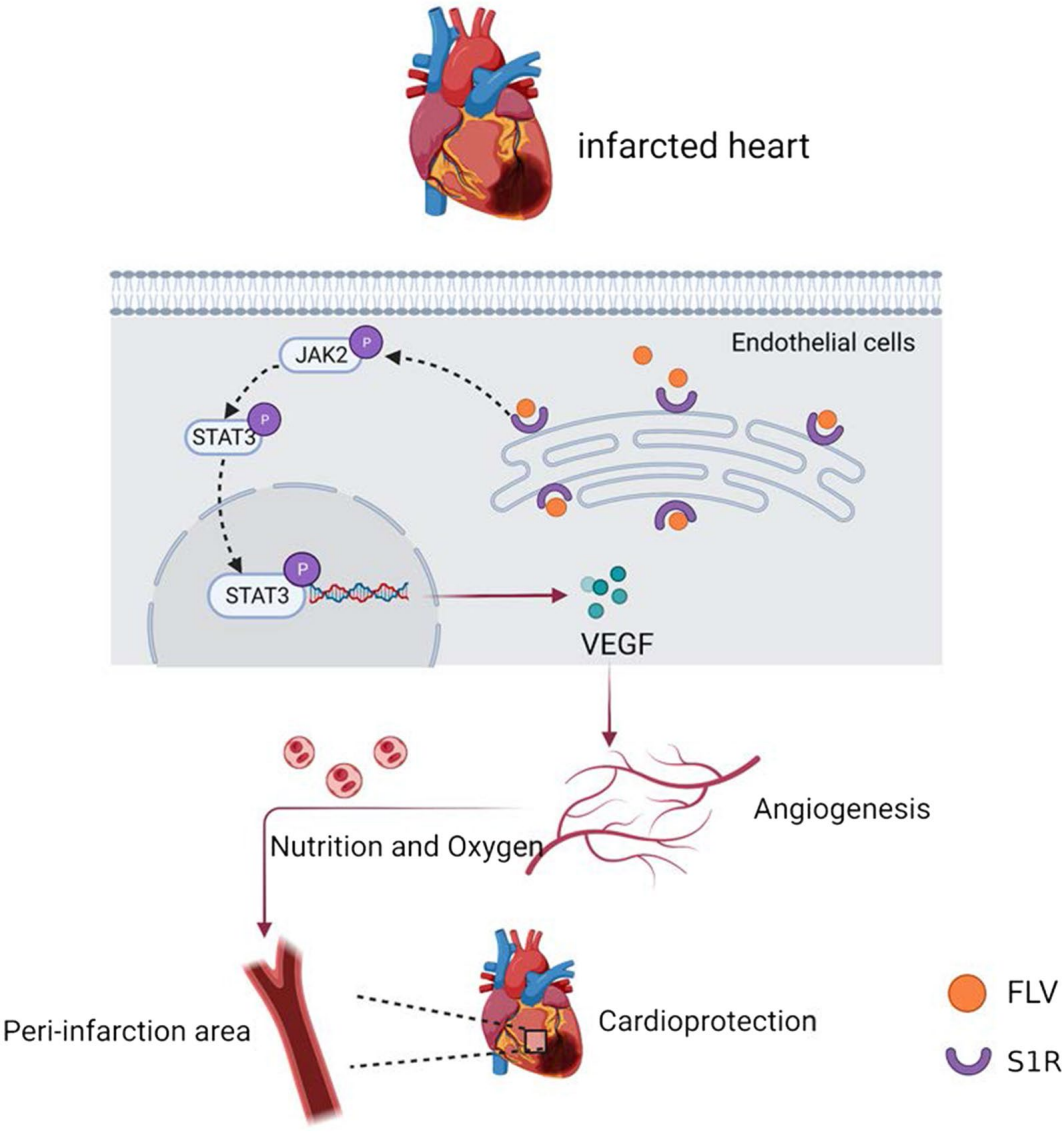
Schematic representation of the angiogenesis effect of S1R stimulation in the PIHF rats. Stimulation of S1R exerts protective effect in PIHF, which were associated with augmentation of microvascular density in peri-infarct region through activation of the JAK2/STAT3 pathway

In this study, we demonstrated that stimulation of S1R facilitated angiogenesis by activation of the JAK2/STAT3 signaling pathway, which further alleviated remodeling factors encompassing interstitial fibrosis and cardiomyocyte apoptosis, followed by cardiac function improvement and slowed down the progression of PIHF (Fig. 7).

### S1R plays putative favorable roles on HF

HF is a severe public health problem, the prevalence of which was increased annually accompanied by overly poor prognosis, therefore, exploitation of an effective therapeutic approach is particularly vital. Accumulating studies have established that S1R is abundantly expressed in cardiac tissue with a reason to speculate potentially protective effect on HF. S1R can mediate the antihypertrophic effect by activating the Akt/eNOS signaling pathway, thus delivering cardioprotection in the TAC-induced HF model (Shinoda et al. 2016). In addition, there is an interaction between the activated S1R and IP_3_R, which can regulate calcium ions flux in mitochondria, thereby enhanced the energic metabolism of cardiomyocytes to maintain physiological function (Sánchez-Alonso et al. 2018). Therefore, stimulation of S1R has cardioprotective effects with certainty, so did the observations from the present study, in which echocardiography and hemodynamic tests, assessing cardiac function and ventricular structural changes, respectively, were significantly ameliorated by S1R, followed by reduction of cardiomyocyte apoptosis and interstitial collagen deposition, demonstrating that S1R was sufficient to protect heart from long-term stimuli. Moreover, all these effects were reversed by administration of the S1R inhibitor BD1047.

### Pro-angiogenic efficacy of stimulation of S1R ameliorates HF

In addition to several factors, including oxidative stress, inflammation and apoptosis, known to exacerbate cardiac function and remodeling, a plethora of studies have established the concept that the insufficiency or malfunction of angiogenesis also facilitates the development of HF (Duan et al. 2015; Bhuiyan et al. 2013). In the proliferative and repairing phase, angiogenesis can increase microvascular perfusion to the injured myocardium, thus, exerting anti-interstitial fibrosis and anti-cardiomyocyte apoptosis effects (Hedman et al. 2003), with abatement of manifestations of HF supervening. Numerous experiments have been conducted to inspect the role for angiogenesis on HF, where the utilization of pro-angiogenesis agents for ischemic myocardial diseases have drawn increasing attention. Moreover, an increasing number of gene targets and regulatory factors have also been identified and scrutinized in clinical trials, such as VEGF_165_ gene therapy (Gyöngyösi et al. 2005; Kastrup et al. 2005; Schumacher et al. 1998) and essential fibroblast growth factor application (Olivieri et al. 2016).

However, contrary to our expectations, the large-scale clinical application of therapeutic angiogenesis agents remained a significant challenge due to the dose, dosing time, delivery route, and carrier selection that have yet to be resolved, for which the identification of pro-angiogenesis targets requires further exploration. Deletion of S1R functions has been implicated with anti-angiogenic activity which reduced HUVECs’ survival (Crottès et al. 2016), moreover, S1R promotes the hERVG/β1-integrin signaling pathway to enhance the aggressive and pro-angiogenic capabilities of tumor cells (Niu et al. 2002). These studies imply pro-angiogenic effect of S1R, with analogous scrutiny in a model of HF absent, in our knowledge. To determine the effect of S1R on angiogenesis in HF rats, the expression of CD31 was detected by immunohistochemical staining in the heart where S1R increased CD31 immunoactivity in the peri-infarct region, so we are the first to confirm that S1R can improve cardiac function by promoting angiogenesis. We further cultured HUVECs with ISO-containing medium to mimic HF pathology which significantly inhibited the proliferation and tube formation of HUVECs, whereas transfection HUVECs with Ad-S1R restored these abilities. These results demonstrated that S1R could exert cardioprotective effects by improving microvascular perfusion to injured myocardium through enhanced angiogenesis, thereby reducing myocardial apoptosis and interstitial fibrosis, so we conjecture S1R may serve as a new target for the treatment of HF in the future.

### S1R activates angiogenesis by regulating the JAK2/STAT3 pathway in HF

The JAK2/STAT3 pathway play a critical role on cardiac angiogenesis in ischemic cardiovascular disease. Endothelial cells can secrete vascular endothelial growth factor (VEGF), a member of a family of growth factors that promote angiogenesis, and, vice versa, act as a target of VEGF. VEGF promotes the proliferation and migration of vascular endothelial cells, a process mediated mainly by binding to VEGFR2 and promoting its phosphorylation (Sun et al. 2018). This procedure is essential for stimulating angiogenesis. Activated STAT3 has been proven to directly bind to the VEGF promoter to promote its expression in tumors (Wei et al. 2003; Sui et al. 2019). Similarly, JAK2/STAT3 activation or overexpression by adenoviral transfection of caSTAT3 into cardiomyocytes or transgenic mice construction can promote myocardial VEGF expression to regulate angiogenesis resulting in causal cardioprotection, which has been observed in MI, ischemia–reperfusion injury, HF, and other cardiac stress conditions (Sui et al. 2019; Dumont et al. 2000; Osugi et al. 2002). Consistently, we shed light on that S1R stimulation can increase angiogenesis by activating the JAK2/STAT3 pathway and that this effect can be antagonized by the use of the S1R inhibitor BD1047. In the present study, establishment of the PIHF rat model resulted in slight activation of JAK2/STAT3, with an insufficiency to protect against the progress of PIHF, and the protein expression of p-JAK2 and p-STAT3 was further upregulated by S1R agonist FLV, demonstrating that stimulate of S1R plays a cardioprotective role through JAK2/STAT3 pathway. However, the effect of S1R on the JAK2/STAT3 pathway might be direct, or indirect as just an association, with the exact regulatory mechanisms remains unelucidated, thus, we exploited specific inhibitor of the JAK2/STAT3 pathway, AG490, to clarify the role of S1R in vitro. In control cells, the JAK2/STAT3 pathway was not activated, nor altered by the administration of Ad-S1R and AG490, in addition to unchanged proliferation and tube-forming capacity of endothelial cells. However, the use of Ad-S1R restored the angiogenesis capacity of HUVECs in the setting of HF, which was diminished after the application of AG490.

### Limitation

The present study still has a few limitations. We used the JAK2/STAT3 pathway inhibitor AG490 rather than using JAK2 or STAT3 siRNA or adenovirus transfection to block its activation. In addition, angiogenesis is a complex regulatory process in which the balance of pro-and anti-angiogenic regulation and whether the JAK2/STAT3 pathway can be altered by influencing anti-angiogenic factors still needs further study.

## Conclusion

In summary, we demonstrated that S1R could act as a pro-angiogenic agent via activation of JAK2/STAT3 pathway on endothelial cells, followed by augmented myocardial perfusion to improve cardiac function and ventricular remodeling after MI.

## Supplementary Information

**Additional file 1.** Supplement figures and tables.

## Data Availability

The datasets used and/or analyzed during the current study are available from the corresponding author on reasonable request.

## Abbreviations

PIHF: Post-infarction heart failure
HF: Heart failure
MI: Myocardial infarction
S1R: Sigma-1 receptor
ROS: Reactive oxygen species
ER: Endoplasmic reticulum
MAM: Mitochondria-associated ER membrane
SSRIs: Several selective serotonin reuptake inhibitors
FLV: Fluvoxamine
ISO: Isoprenaline
LAD: Left coronary artery
VEGF: Vascular endothelial growth factor

## References

[CR1] Abdullah CS, Alam S, Aishwarya R, Miriyala S, Panchatcharam M, Bhuiyan MAN (2018). Cardiac dysfunction in the sigma 1 receptor knockout mouse associated with impaired mitochondrial dynamics and bioenergetics. J Am Heart Assoc.

[CR2] Beohar N, Rapp J, Pandya S, Losordo DW (2010). Rebuilding the damaged heart: the potential of cytokines and growth factors in the treatment of ischemic heart disease. J Am Coll Cardiol.

[CR3] Bhuiyan MS, Tagashira H, Fukunaga K (2013). Crucial interactions between selective serotonin uptake inhibitors and sigma-1 receptor in heart failure. J Pharmacol Sci.

[CR4] Chang X, Lochner A, Wang HH, Wang S, Zhu H, Ren J (2021). Coronary microvascular injury in myocardial infarction: perception and knowledge for mitochondrial quality control. Theranostics.

[CR5] Chen Z, Han ZC (2008). STAT3: a critical transcription activator in angiogenesis. Med Res Rev.

[CR6] Cochain C, Channon KM, Silvestre JS (2013). Angiogenesis in the infarcted myocardium. Antioxid Redox Signal.

[CR7] Crottès D, Rapetti-Mauss R, Alcaraz-Perez F, Tichet M, Gariano G, Martial S (2016). SIGMAR1 regulates membrane electrical activity in response to extracellular matrix stimulation to drive cancer cell invasiveness. Can Res.

[CR8] Duan Q, Yang L, Gong W, Chaugai S, Wang F, Chen C (2015). MicroRNA-214 is upregulated in heart failure patients and suppresses XBP1-mediated endothelial cells angiogenesis. J Cell Physiol.

[CR9] Dumont EA, Hofstra L, van Heerde WL, van den Eijnde S, Doevendans PA, DeMuinck E (2000). Cardiomyocyte death induced by myocardial ischemia and reperfusion: measurement with recombinant human annexin-V in a mouse model. Circulation.

[CR10] Eelen G, Treps L, Li X, Carmeliet P (2020). Basic and therapeutic aspects of angiogenesis updated. Circ Res.

[CR11] Fo Y, Zhang C, Chen X, Liu X, Ye T, Guo Y (2020). Chronic sigma-1 receptor activation ameliorates ventricular remodeling and decreases susceptibility to ventricular arrhythmias after myocardial infarction in rats. Eur J Pharmacol.

[CR12] Fraccarollo D, Galuppo P, Hildemann S, Christ M, Ertl G, Bauersachs J (2003). Additive improvement of left ventricular remodeling and neurohormonal activation by aldosterone receptor blockade with eplerenone and ACE inhibition in rats with myocardial infarction. J Am Coll Cardiol.

[CR13] Gabriel-Costa D (2018). The pathophysiology of myocardial infarction-induced heart failure. Pathophysiology.

[CR14] Gyöngyösi M, Khorsand A, Zamini S, Sperker W, Strehblow C, Kastrup J (2005). NOGA-guided analysis of regional myocardial perfusion abnormalities treated with intramyocardial injections of plasmid encoding vascular endothelial growth factor A-165 in patients with chronic myocardial ischemia: subanalysis of the EUROINJECT-ONE multicenter double-blind randomized study. Circulation.

[CR15] Hashimoto K (2013). Sigma-1 receptor chaperone and brain-derived neurotrophic factor: emerging links between cardiovascular disease and depression. Prog Neurobiol.

[CR16] Hayashi T, Su TP (2007). Sigma-1 receptor chaperones at the ER-mitochondrion interface regulate Ca(2+) signaling and cell survival. Cell.

[CR17] Hedman M, Hartikainen J, Syvänne M, Stjernvall J, Hedman A, Kivelä A (2003). Safety and feasibility of catheter-based local intracoronary vascular endothelial growth factor gene transfer in the prevention of postangioplasty and in-stent restenosis and in the treatment of chronic myocardial ischemia: phase II results of the Kuopio Angiogenesis Trial (KAT). Circulation.

[CR18] Jenča D, Melenovský V, Stehlik J, Staněk V, Kettner J, Kautzner J (2021). Heart failure after myocardial infarction: incidence and predictors. ESC Heart Failure.

[CR19] Kastrup J, Jørgensen E, Rück A, Tägil K, Glogar D, Ruzyllo W (2005). Direct intramyocardial plasmid vascular endothelial growth factor-A165 gene therapy in patients with stable severe angina pectoris A randomized double-blind placebo-controlled study: the Euroinject One trial. J Am Coll Cardiol.

[CR20] Khurana R, Simons M, Martin JF, Zachary IC (2005). Role of angiogenesis in cardiovascular disease: a critical appraisal. Circulation.

[CR21] Liang B, Zhao YX, Zhang XX, Liao HL, Gu N (2020). Reappraisal on pharmacological and mechanical treatments of heart failure. Cardiovasc Diabetol.

[CR22] Lou L, Li C, Wang J, Wu A, Zhang T, Ma Z (2021). Yiqi Huoxue preserves heart function by upregulating the Sigma-1 receptor in rats with myocardial infarction. Exp Ther Med.

[CR23] Moghiman T, Barghchi B, Esmaeili SA, Shabestari MM, Tabaee SS, Momtazi-Borojeni AA (2021). Therapeutic angiogenesis with exosomal microRNAs: an effectual approach for the treatment of myocardial ischemia. Heart Fail Rev.

[CR24] Niu G, Wright KL, Huang M, Song L, Haura E, Turkson J (2002). Constitutive Stat3 activity up-regulates VEGF expression and tumor angiogenesis. Oncogene.

[CR25] Olivieri M, Amata E, Vinciguerra S, Fiorito J, Giurdanella G, Drago F (2016). Antiangiogenic effect of (±)-haloperidol metabolite II valproate ester [(±)-MRJF22] in human microvascular retinal endothelial cells. J Med Chem.

[CR26] Osugi T, Oshima Y, Fujio Y, Funamoto M, Yamashita A, Negoro S (2002). Cardiac-specific activation of signal transducer and activator of transcription 3 promotes vascular formation in the heart. J Biol Chem.

[CR27] Qu J, Li M, Li D, Xin Y, Li J, Lei S (2021). Stimulation of sigma-1 receptor protects against cardiac fibrosis by alleviating IRE1 pathway and autophagy impairment. Oxid Med Cell Longev.

[CR28] Robson MJ, Turner RC, Naser ZJ, McCurdy CR, O'Callaghan JP, Huber JD (2014). SN79, a sigma receptor antagonist, attenuates methamphetamine-induced astrogliosis through a blockade of OSMR/gp130 signaling and STAT3 phosphorylation. Exp Neurol.

[CR29] Roger VL (2013). Epidemiology of heart failure. Circ Res.

[CR30] Sánchez-Alonso S, Alcaraz-Serna A, Sánchez-Madrid F, Alfranca A (2018). Extracellular vesicle-mediated immune regulation of tissue remodeling and angiogenesis after myocardial infarction. Front Immunol.

[CR31] Savarese G, Lund LH (2017). Global public health burden of heart failure. Card Fail Rev.

[CR32] Scarabelli TM, Knight R, Stephanou A, Townsend P, Chen-Scarabelli C, Lawrence K (2006). Clinical implications of apoptosis in ischemic myocardium. Curr Probl Cardiol.

[CR33] Schumacher B, Pecher P, von Specht BU, Stegmann T (1998). Induction of neoangiogenesis in ischemic myocardium by human growth factors: first clinical results of a new treatment of coronary heart disease. Circulation.

[CR34] Shinoda Y, Tagashira H, Bhuiyan MS, Hasegawa H, Kanai H, Fukunaga K (2016). Haloperidol aggravates transverse aortic constriction-induced heart failure via mitochondrial dysfunction. J Pharmacol Sci.

[CR35] Spears JR (2019). Reperfusion microvascular ischemia after prolonged coronary occlusion: implications and treatment with local supersaturated oxygen delivery. Hypoxia (auckland, NZ).

[CR36] Sui YB, Wang Y, Liu L, Liu F, Zhang YQ (2019). Astragaloside IV alleviates heart failure by promoting angiogenesis through the JAK-STAT3 pathway. Pharm Biol.

[CR37] Sun J, Huang W, Yang SF, Zhang XP, Yu Q, Zhang ZQ (2018). Gαi1 and Gαi3mediate VEGF-induced VEGFR2 endocytosis, signaling and angiogenesis. Theranostics.

[CR38] Tabibiazar R, Rockson SG (2001). Angiogenesis and the ischaemic heart. Eur Heart J.

[CR39] Tagashira H, Bhuiyan MS, Shioda N, Fukunaga K (2014). Fluvoxamine rescues mitochondrial Ca2+ transport and ATP production through σ(1)-receptor in hypertrophic cardiomyocytes. Life Sci.

[CR40] Vahabzadeh G, Soltani H, Barati M, Golab F, Jafari-Sabet M, Safari S (2020). Noscapine protects the H9c2 cardiomyocytes of rats against oxygen-glucose deprivation/reperfusion injury. Mol Biol Rep.

[CR41] van der Meer P, Gaggin HK, Dec GW (2019). ACC/AHA versus ESC guidelines on heart failure: JACC guideline comparison. J Am Coll Cardiol.

[CR42] Wang Y, Zhang X, Fu Y, Fu D, Zhen D, Xing A (2021). 1, 8-cineole protects against ISO-induced heart failure by inhibiting oxidative stress and ER stress in vitro and in vivo. Eur J Pharmacol.

[CR43] Wei D, Le X, Zheng L, Wang L, Frey JA, Gao AC (2003). Stat3 activation regulates the expression of vascular endothelial growth factor and human pancreatic cancer angiogenesis and metastasis. Oncogene.

[CR44] Weiwei C, Runlin G, Lisheng L, Manlu Z, Wen W, Yongjun W (2016). Outline of the report on cardiovascular diseases in China, 2014. Eur Heart J Suppl.

[CR45] Wu X, Reboll MR, Korf-Klingebiel M, Wollert KC (2021). Angiogenesis after acute myocardial infarction. Cardiovasc Res.

[CR46] Zhao XB, Qin Y, Niu YL, Yang J (2018). Matrine inhibits hypoxia/reoxygenation-induced apoptosis of cardiac microvascular endothelial cells in rats via the JAK2/STAT3 signaling pathway. Biomed Pharmacother.

