# Supplementary material for: Activation of the sigma-1 receptor exerts cardioprotection in a rodent model of chronic heart failure by stimulation of angiogenesis
Source: Mol Med. 2022 Aug 3;28:87. doi: 10.1186/s10020-022-00517-1 (PMC9347174; doi:10.1186/s10020-022-00517-1)
Supplement: Supplementary file 1 — Additional file 1. Supplement figures and tables. [file 10020_2022_517_MOESM1_ESM.docx]

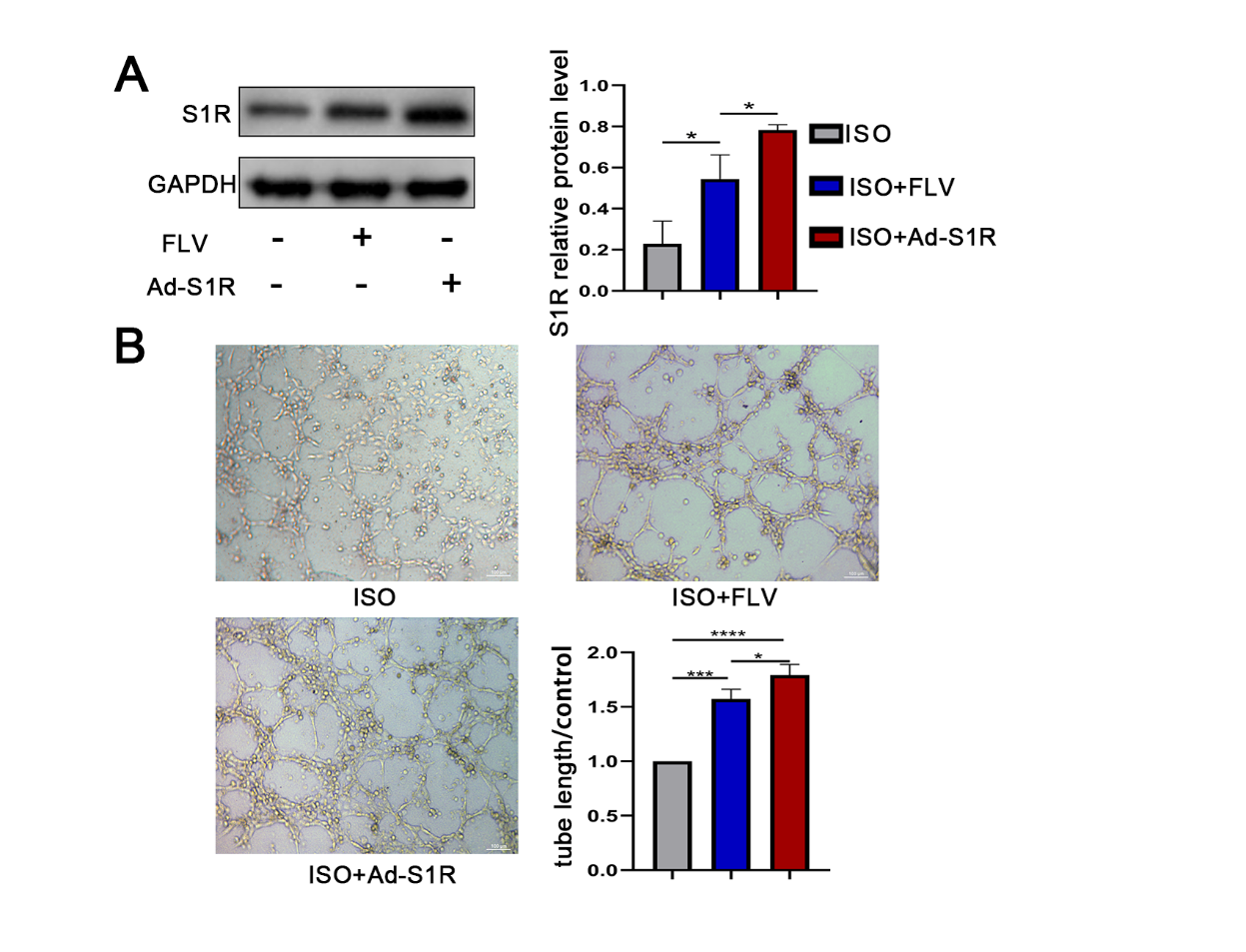


**SUPPLEMENTARY FIGURE.1** Application of FLV and transfection of Ad-S1R both promoted angiogenesis of HUVECs. A Western blot results showed that application of FLV and transfection of Ad-S1R upregulated the sigma-1 receptor expression. N = 3 for quantified analysis; B Application of FLV and transfection of Ad-S1R facilitated the tube-forming ability of HUVECs. *: p<0.05; **: p<0.01; ***: p<0.005; ****: p<0.001

**
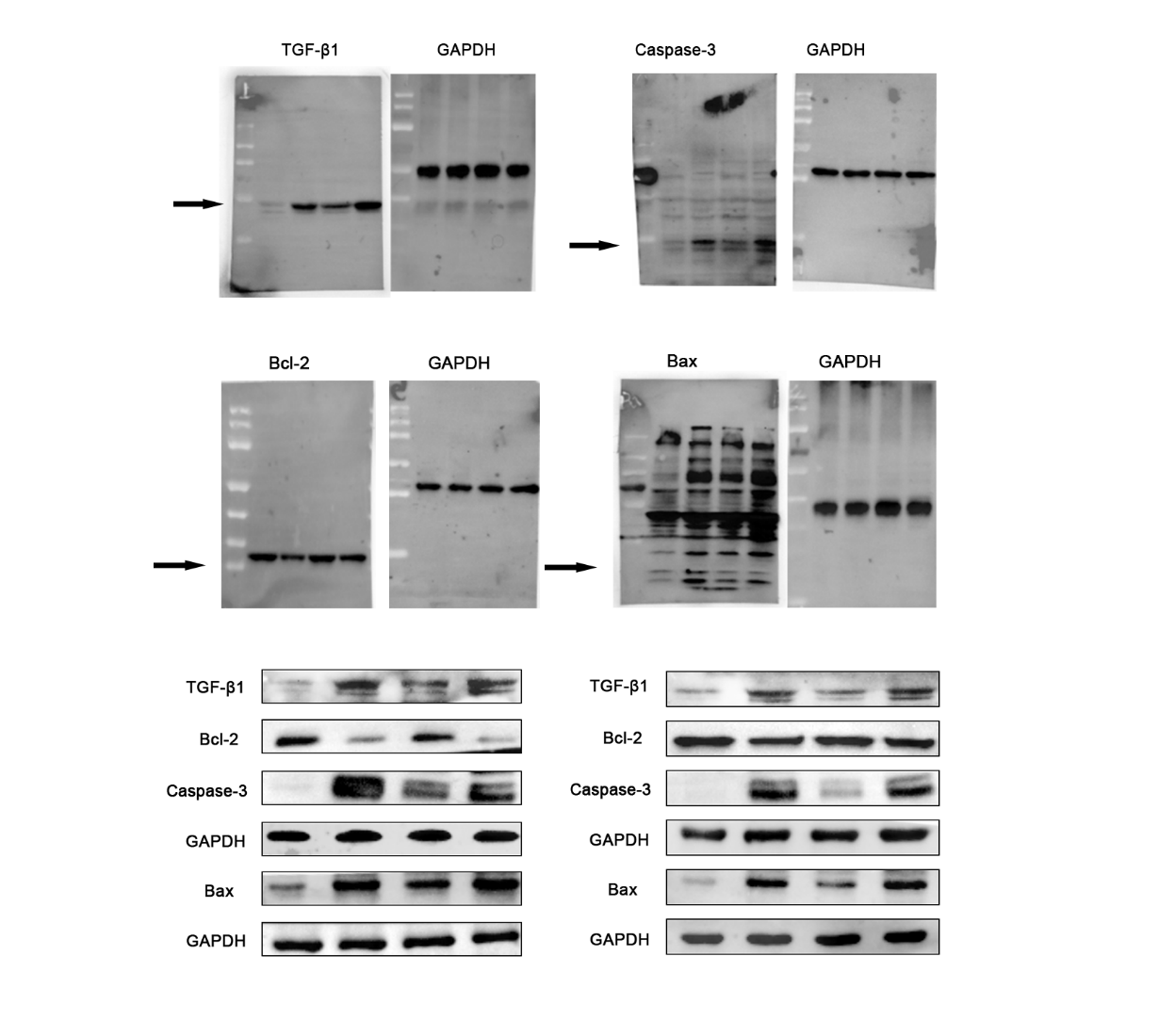
 SUPPLEMENTARY FIGURE.2** The whole western blots results of Manuscript Figure.3C

**
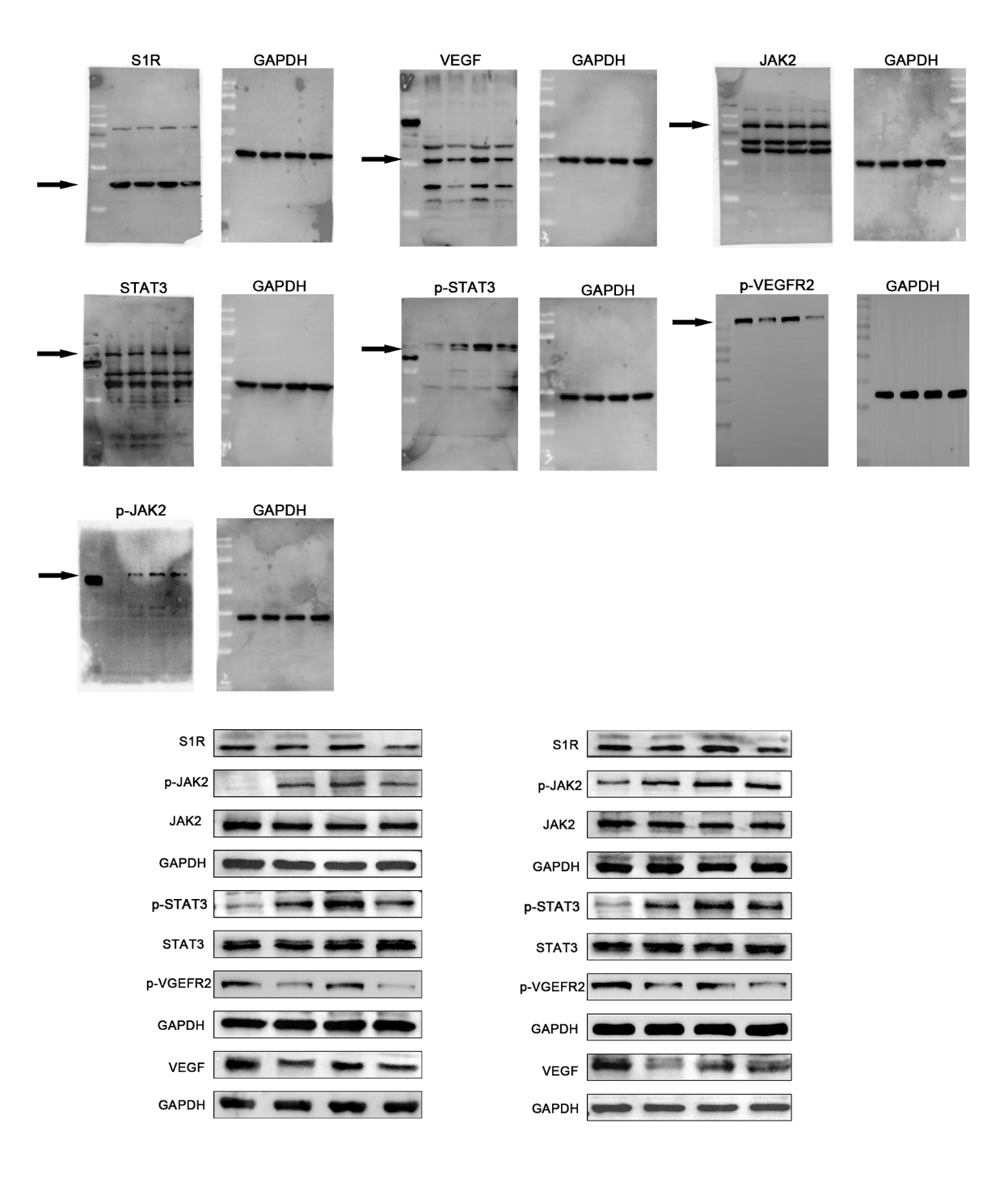
 SUPPLEMENTARY FIGURE.3** The whole western blots results of Manuscript Figure.4E
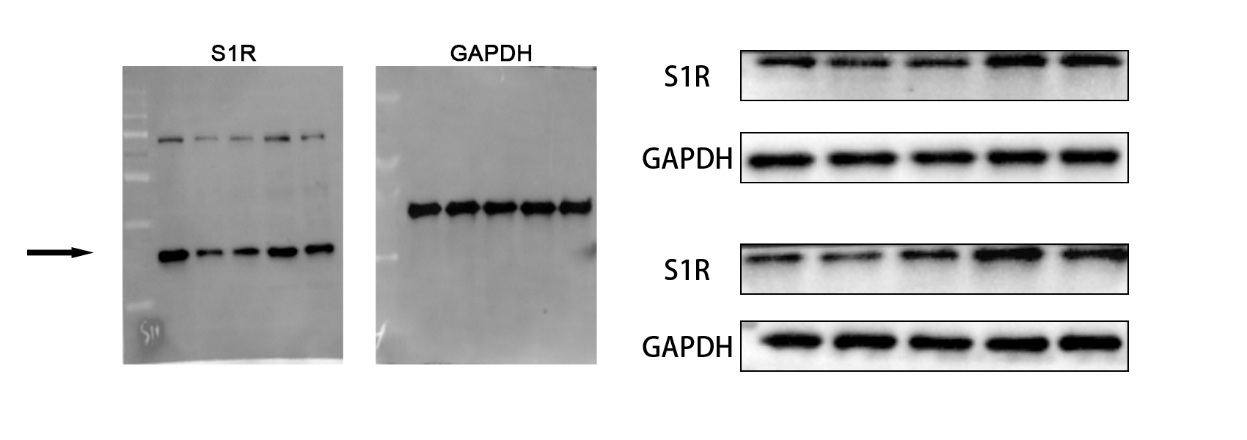


**SUPPLEMENTARY FIGURE.4** The whole western blots results of Manuscript Figure.5A
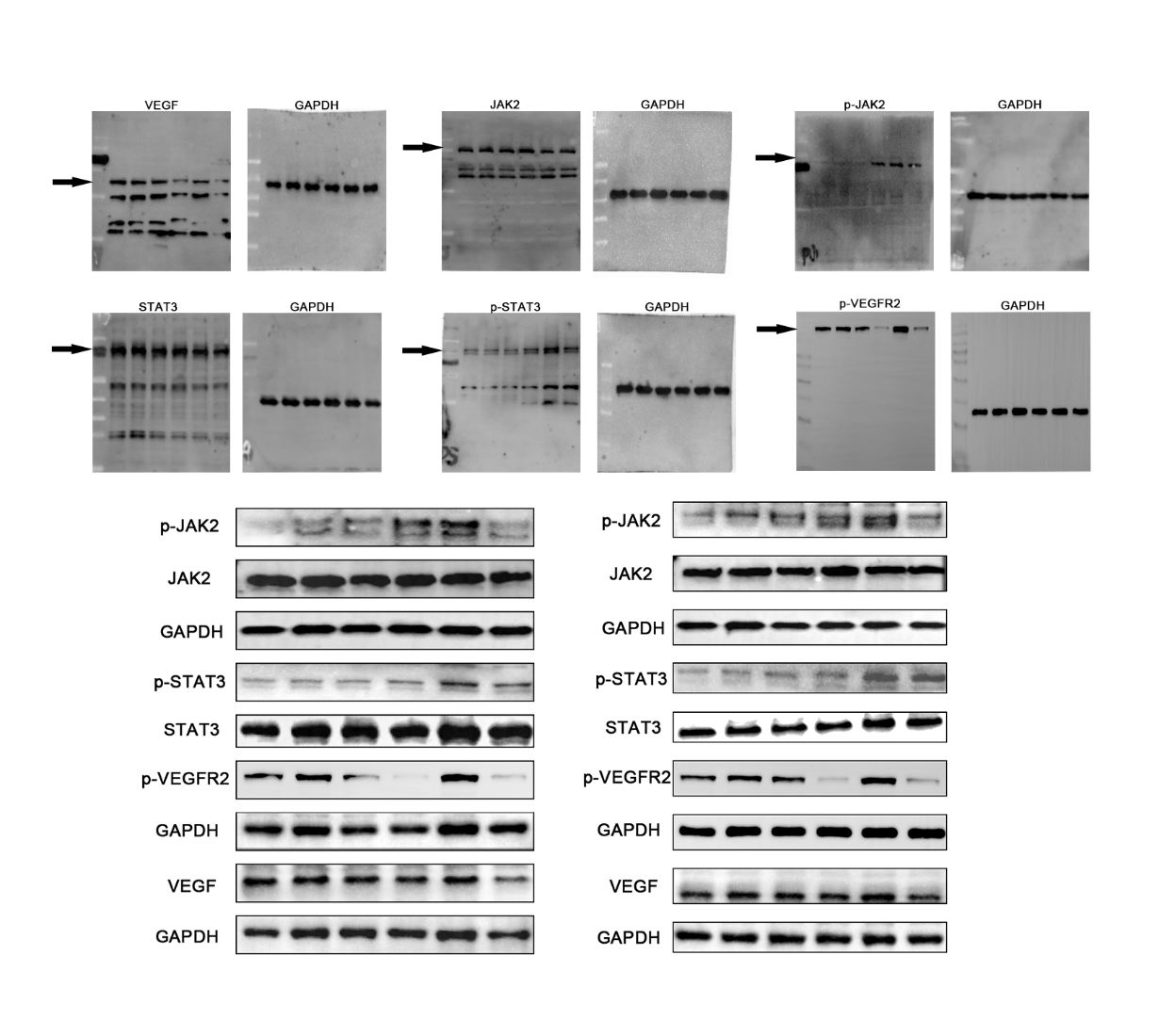
 **SUPPLEMENTARY FIGURE.5** The whole western blots results of Manuscript Figure.6A
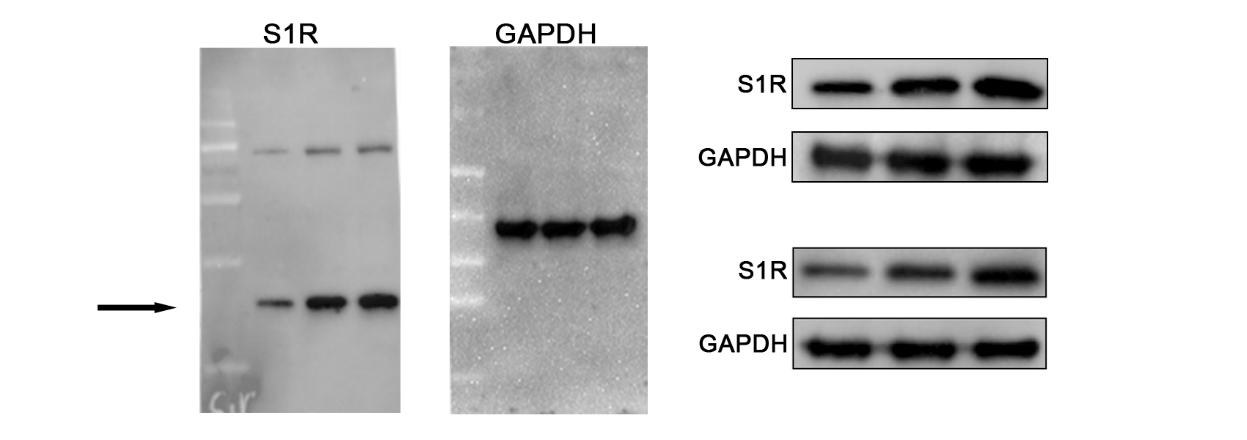


**SUPPLEMENTARY FIGURE.6** The whole western blots results of Supplement Figure.1A


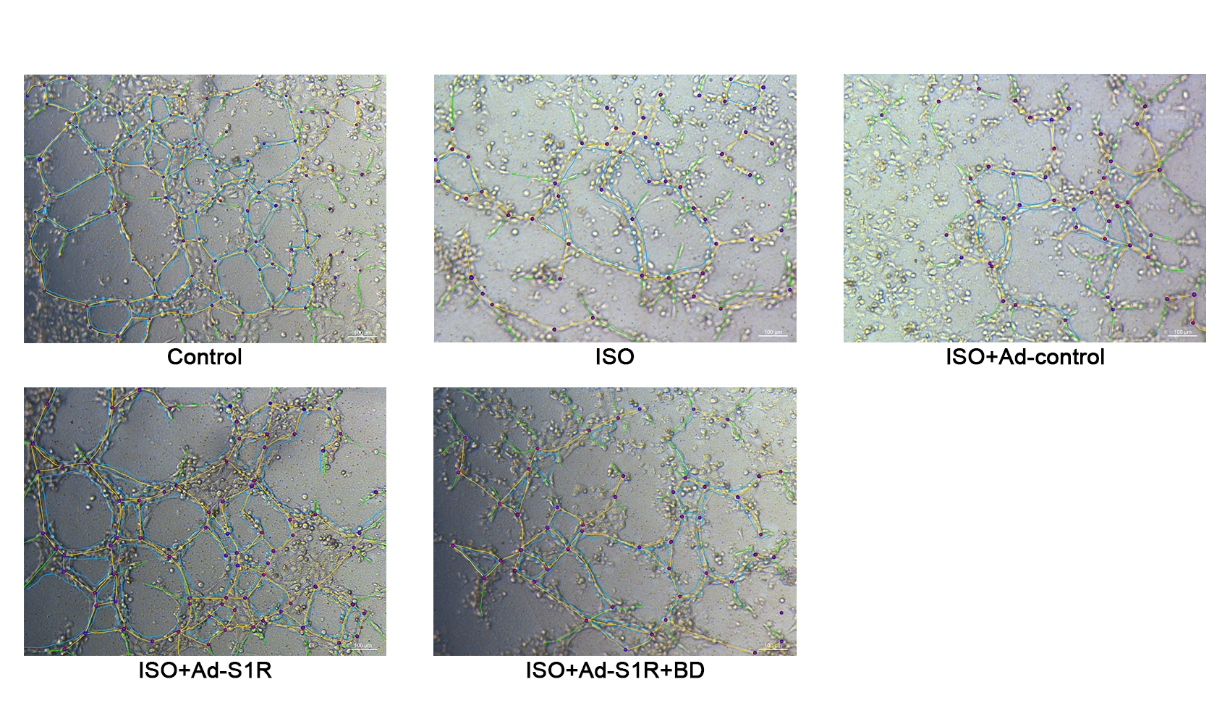


**SUPPLEMENTARY FIGURE.7** The resulting graph of the tube formation analysis of Manuscript Figure.5C. The blue, green, and yellow lines collectively delineated the formed tube lumen, and the pink dots with dark blue border represented the connective points between adjacent tube lumens.

**Table 1** Analytic result of representative tube formation photomicrographs of Figure.5C in the manuscript

|  | Control | ISO | ISO+Ad-ctl | ISO+Ad-S1R | ISO+Ad-S1R+BD |
| --- | --- | --- | --- | --- | --- |
| 1 | 1.027349 | 0.482854 | 0.534221 | 1.232818 | 0.544271 |
| 2 | 0.985147 | 0.452003 | 0.413762 | 0.994999 | 0.507933 |
| 3 | 0.987504 | 0.428146 | 0.447174 | 0.913377 | 0.485232 |


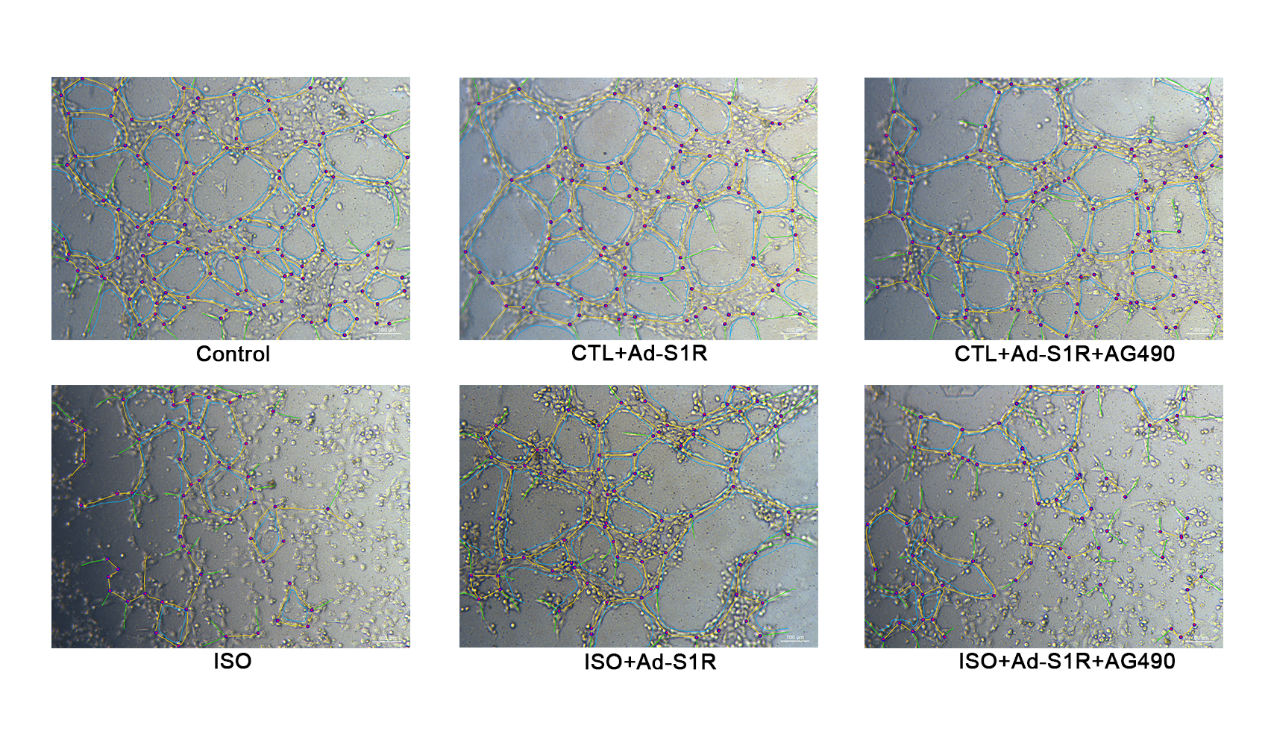


**SUPPLEMENTARY FIGURE.8** The resulting graph of the tube formation analysis of Manuscript Figure.6B. The blue, green, and yellow lines concurrently marked the formed tube lumen out.

**Table 2** Analytic result of representative tube formation photomicrographs of Figure.6B in the manuscript

|  | Control | Ctl+Ad-S1R | Ctl+Ad-S1R+AG490 | ISO | ISO+Ad-S1R | ISO+Ad-S1R+AG490 |
| --- | --- | --- | --- | --- | --- | --- |
| 1 | 1.035702 | 0.952846 | 1.128915 | 0.445352 | 0.921775 | 0.372853 |
| 2 | 1.012958 | 1.114254 | 0.992699 | 0.354535 | 0.942051 | 0.425442 |
| 3 | 0.95134 | 0.913286 | 0.856206 | 0.351996 | 0.827666 | 0.428103 |


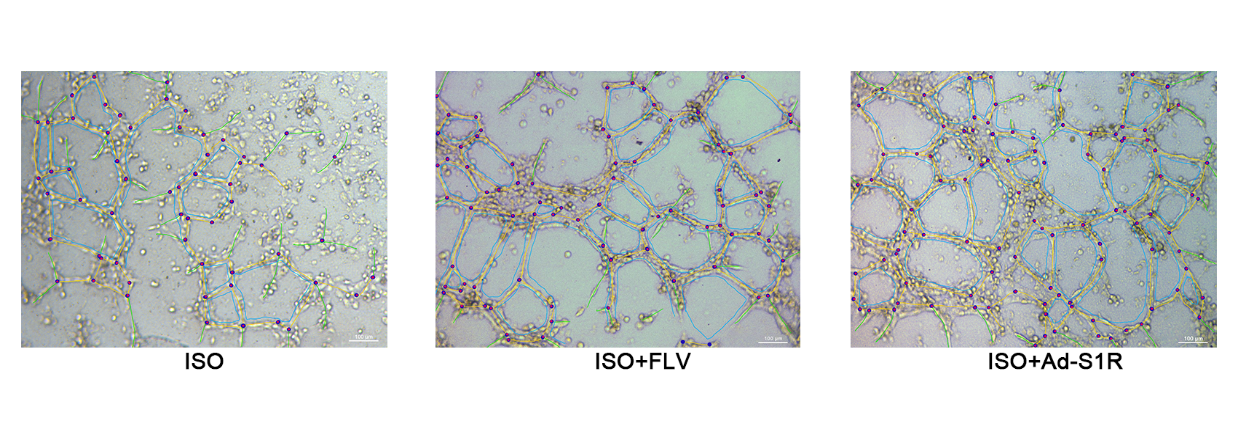


**SUPPLEMENTARY FIGURE.9** The resulting graph of the tube formation analysis of Supplement Figure.1B. The blue, green, and yellow lines collectively delineated the formed tube lumen, and the pink dots with dark blue border represented the connective points between adjacent tube lumens.

**Table 3** Analytic result of representative tube formation photomicrographs of Supplement Figure.1B

|  | ISO | ISO+FLV | ISO+Ad-S1R |
| --- | --- | --- | --- |
| 1 | 1.051175 | 1.636702 | 1.911001 |
| 2 | 0.981347 | 1.51141 | 1.747727 |
| 3 | 0.967478 | 1.477905 | 1.713195 |
